# Supplementary figures and images for: The evolution of mammalian Rem2: unraveling the impact of purifying selection and coevolution on protein function, and implications for human disorders
Source: Front Bioinform. 2024 Jun 24;4:1381540. doi: 10.3389/fbinf.2024.1381540 (PMC11228553; doi:10.3389/fbinf.2024.1381540)

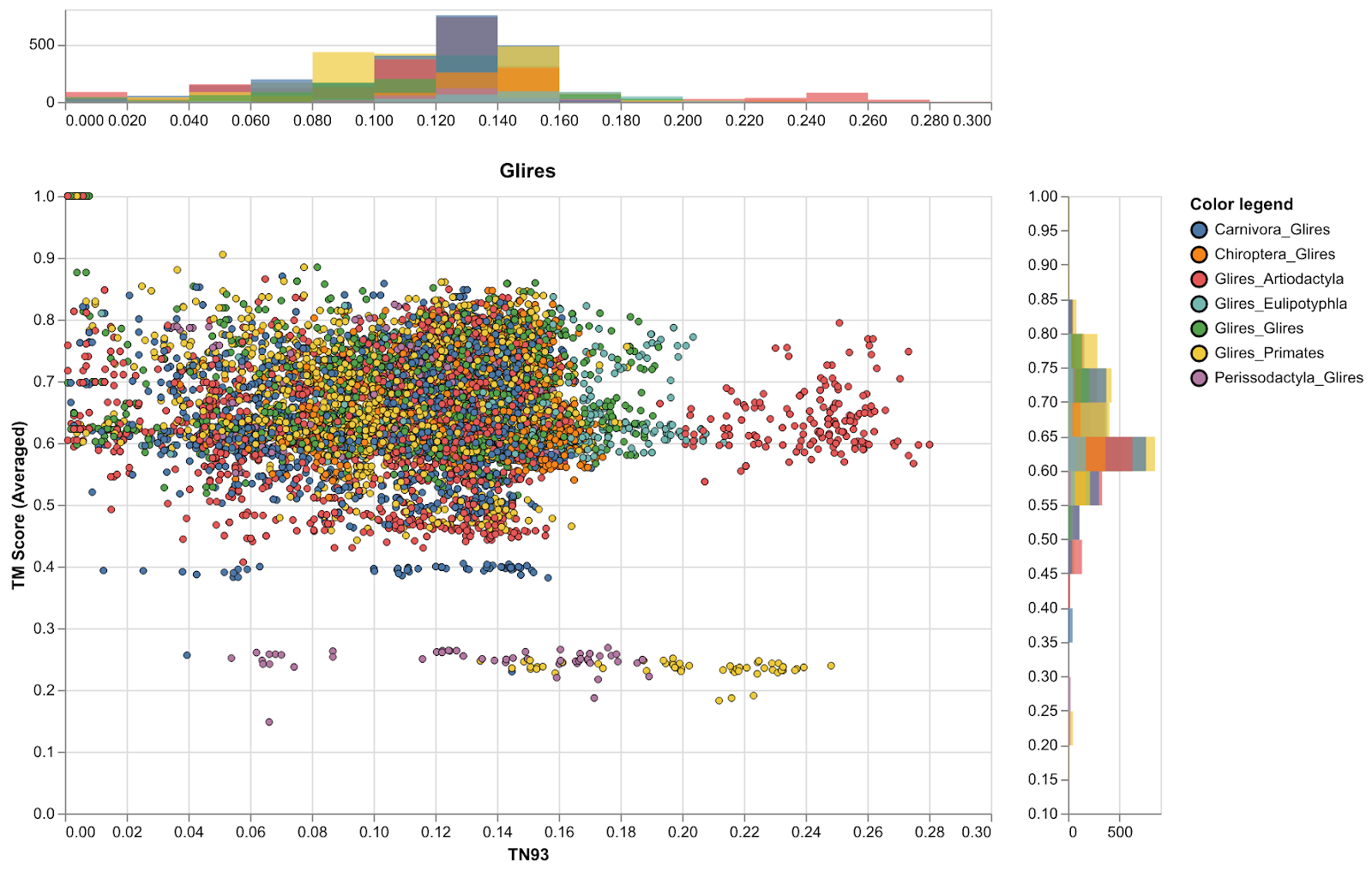

Supplement: Supplementary file 3 [file Image5.png]

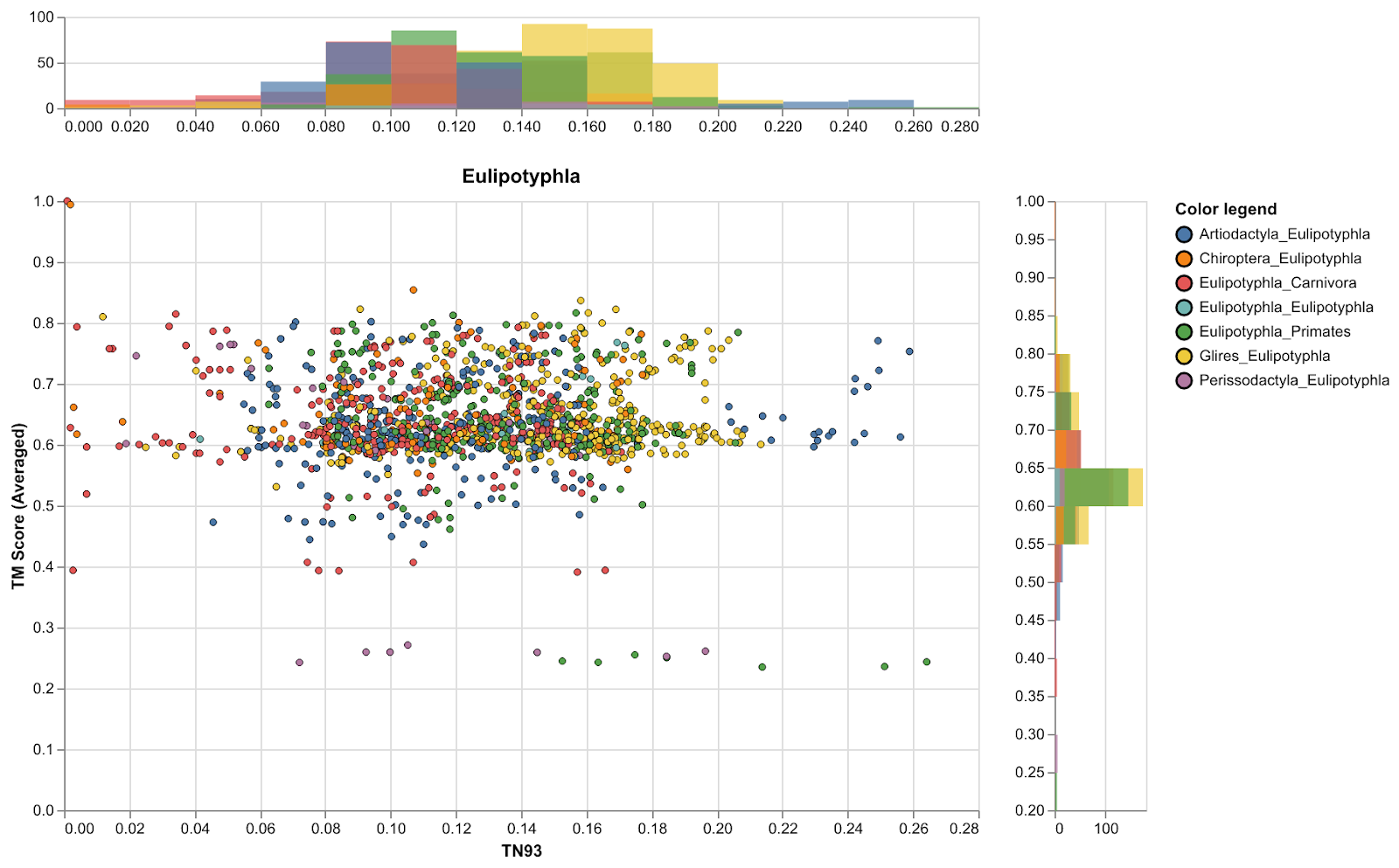

Supplement: Supplementary file 4 [file Image4.png]

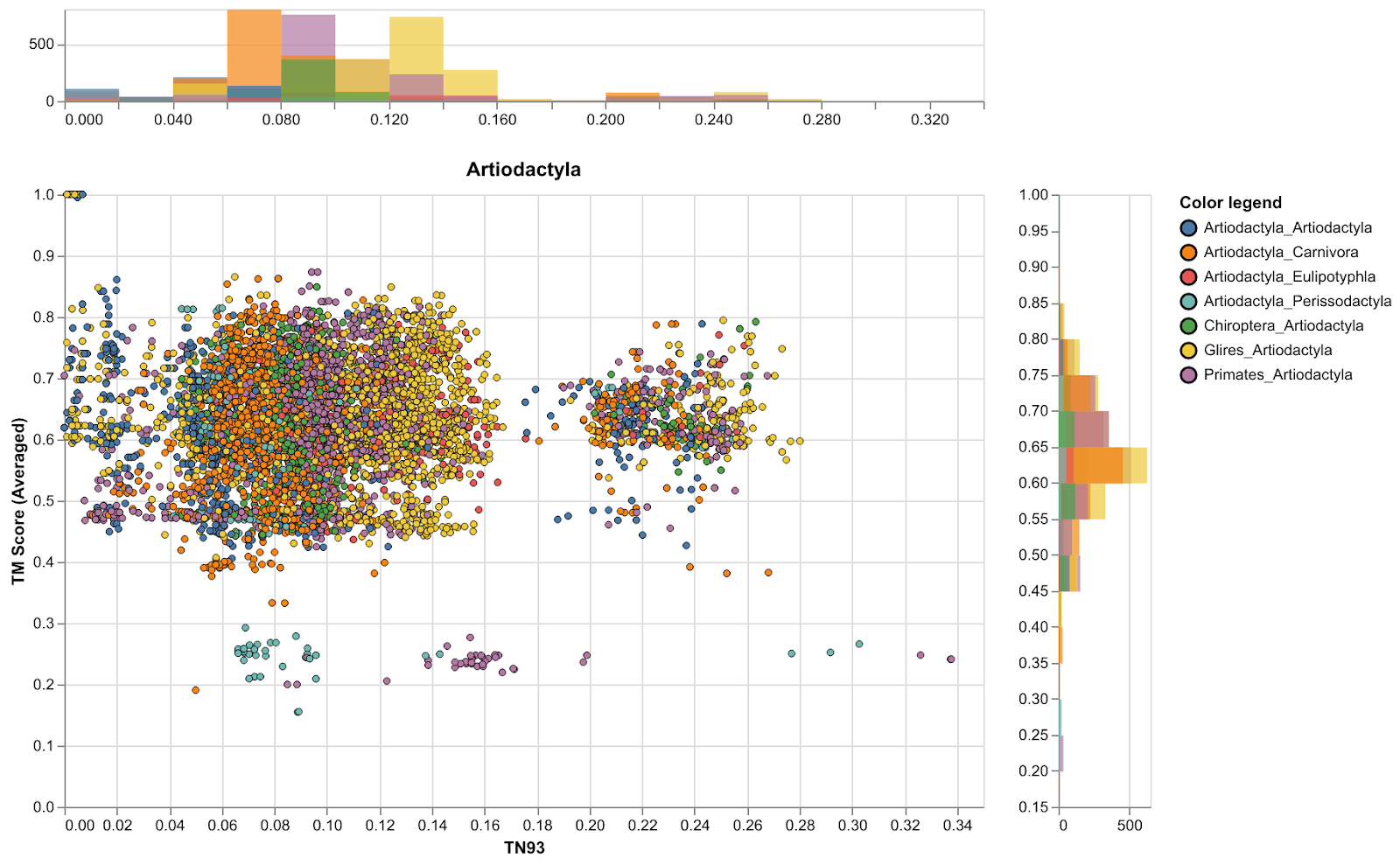

Supplement: Supplementary file 5 [file Image2.png]

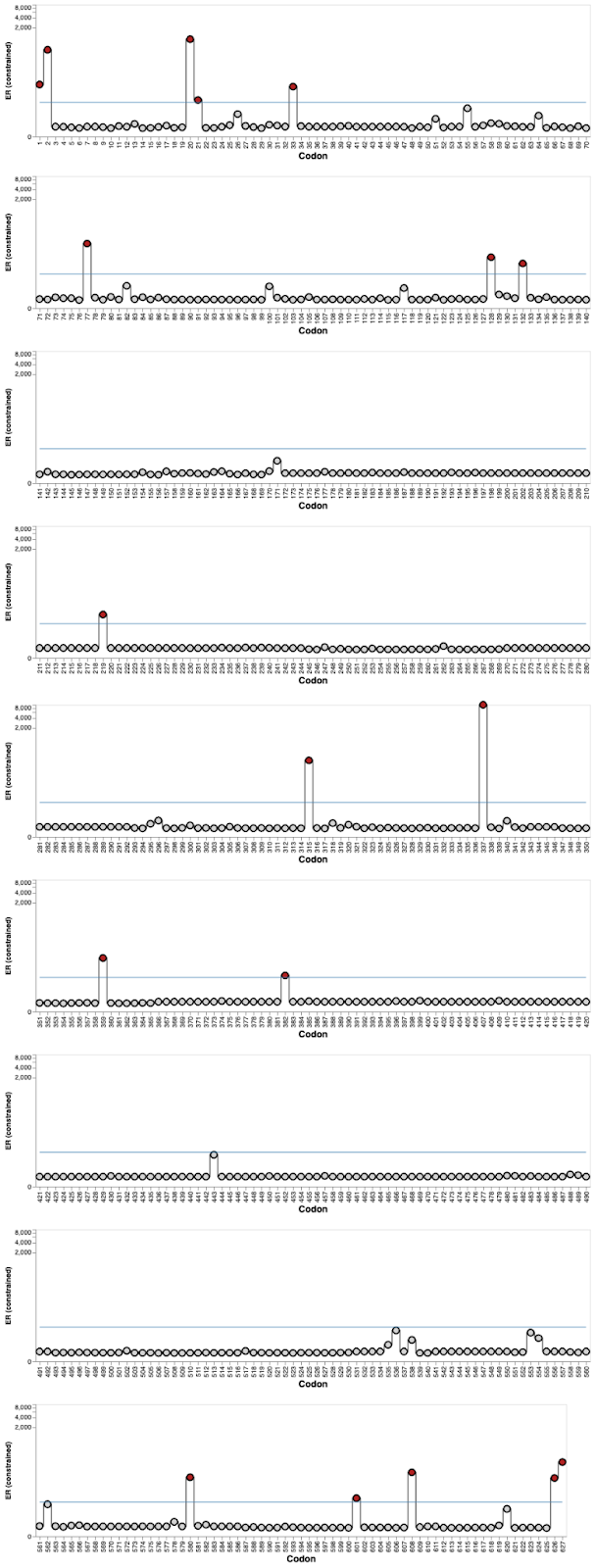

Supplement: Supplementary file 8 [file Image1.png]

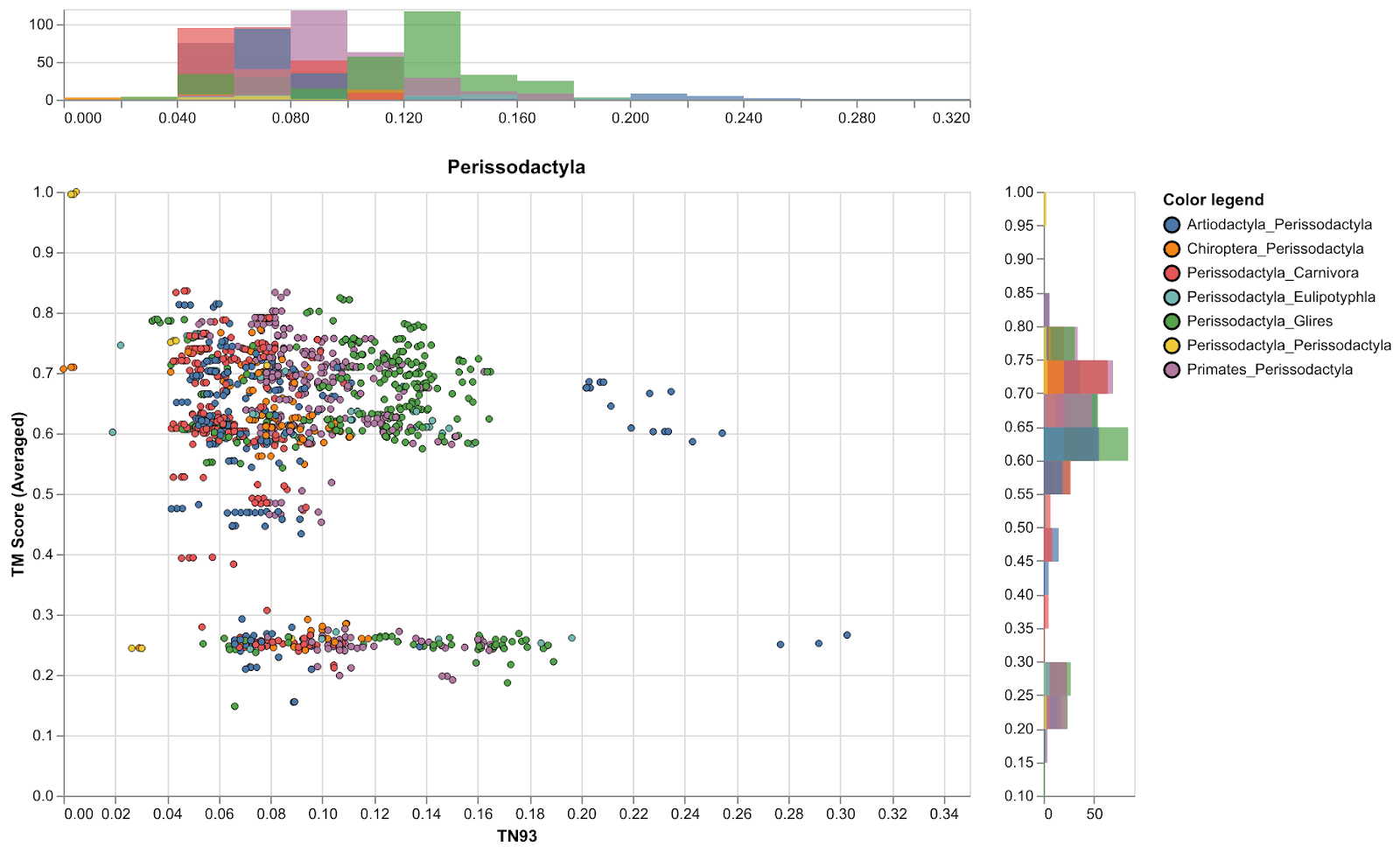

Supplement: Supplementary file 9 [file Image6.png]

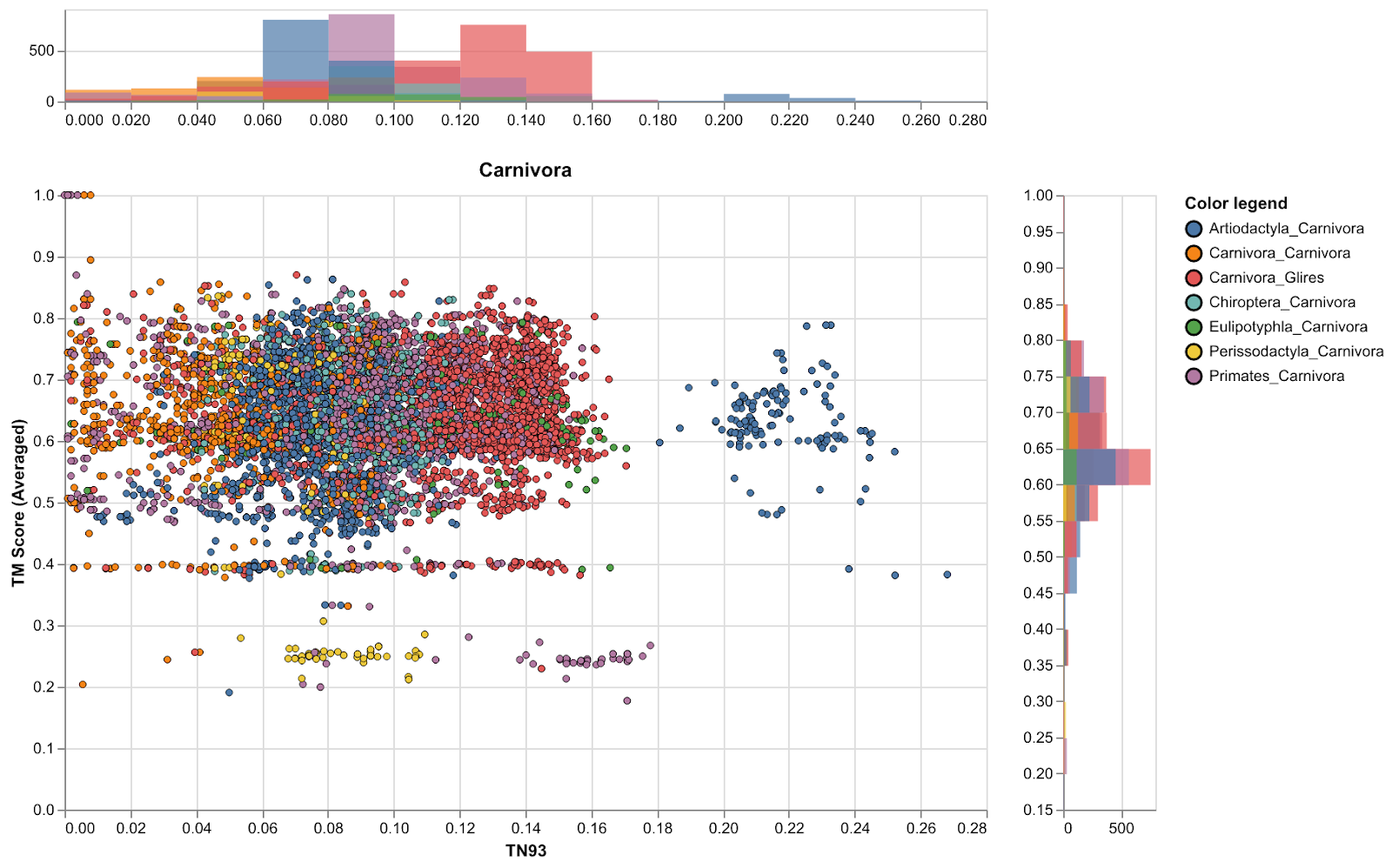

Supplement: Supplementary file 11 [file Image3.png]
